# Recent trends in miRNA therapeutics and the application of plant miRNA for prevention and treatment of human diseases

**DOI:** 10.1186/s43094-022-00413-9

**Published:** 2022-04-01

**Authors:** Atiyabanu N. Saiyed, Abhay R. Vasavada, S. R. Kaid Johar

**Affiliations:** 1 Department of Cell and Molecular Biology, Iladevi Cataract and IOL Research Centre, Ahmedabad, Gujarat India; 2 Ph.D. scholar of Manipal Academy of Higher Education, Manipal, Karnataka India; 3 Department of Zoology, BMTC, Human Genetics, USSC, Gujarat University, Ahmedabad, Gujarat India

**Keywords:** miRNA, Drug, Therapeutics, Cross-kingdom, Gene regulation, Epigenetics, Post-transcription

## Abstract

**Background:**

Researchers now have a new avenue to investigate when it comes to miRNA-based therapeutics. miRNAs have the potential to be valuable biomarkers for disease detection. Variations in miRNA levels may be able to predict changes in normal physiological processes. At the epigenetic level, miRNA has been identified as a promising candidate for distinguishing and treating various diseases and defects.

**Main body:**

In recent pharmacology, plants miRNA-based drugs have demonstrated a potential role in drug therapeutics. The purpose of this review paper is to discuss miRNA-based therapeutics, the role of miRNA in pharmacoepigenetics modulations, plant miRNA inter-kingdom regulation, and the therapeutic value and application of plant miRNA for cross-kingdom approaches. Target prediction and complementarity with host genes, as well as cross-kingdom gene interactions with plant miRNAs, are also revealed by bioinformatics research. We also show how plant miRNA can be transmitted from one species to another by crossing kingdom boundaries in this review. Despite several unidentified barriers to plant miRNA cross-transfer, plant miRNA-based gene regulation in trans-kingdom gene regulation may soon be valued as a possible approach in plant-based drug therapeutics.

**Conclusion:**

This review summarised the biochemical synthesis of miRNAs, pharmacoepigenetics, drug therapeutics and miRNA transkingdom transfer.

## Background

MicroRNA (miRNA) and gene expression regulation have paved the way for new therapeutic approaches. miRNA has the ability to control effects of various types of mutation, gene dysregulation, and incorrect function of cellular, biological, metabolic, and physiological pathways [1, 2].

## Main text

**Fig. 1:**
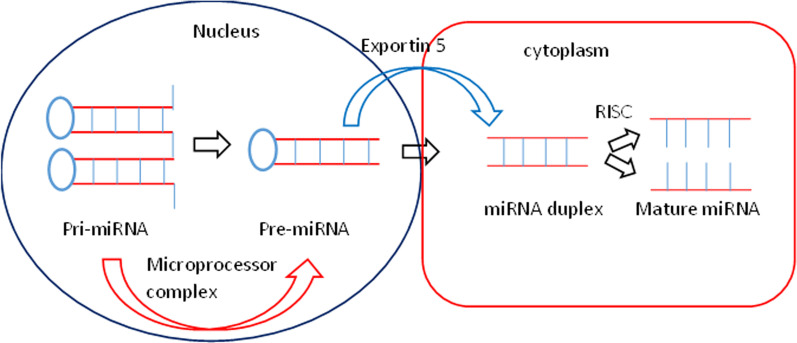
With the help of a microprocessor complex in the nucleus, pri-miRNA is transformed to pre-miRNA. After that, pre-miRNA is transferred to the cytoplasm by exportin 5 and miRNA duplex formation occurs. Then, using RISC assembly, the miRNA duplex was transformed into mature miRNA

miRNA is a class of small non-coding RNAs (ncRNAs) that can be up to 22 nucleotides long and regulate multiple target genes at the post-transcriptional level [3, 4]. The majority of miRNAs are expressed as primary miRNAs (pri-miRNAs), which are transcribed from DNA sequences and can be further processed to become precursor miRNAs (pre-miRNAs) and then mature miRNAs [5] (Fig. 1). Because of their well-known regulatory effects on human diseases, there has been a surge of interest in ncRNA research in the last decade. ncRNAs function post-transcriptionally via messenger RNA (mRNA) disruption [6]. The purpose of this review paper is to understand the progress in miRNA-based therapeutics, discuss the medicinal value of plant-based miRNA and inter-species transfer. This review also discusses the mechanism and function of miRNA and plant-based miRNA in disease management. We have summarised key aspects of the experimental and computational methods used to evaluate the therapeutic value of miRNA and natural compound-based miRNA.

## miRNA biochemical synthesis

miRNAs regulate biological processes such as cell growth, death, development, and differentiation [7]. According to various studies, miRNA travel between different regions of the cell to estimate the translation and transcription processes [8]. It has the potential to act as a guide molecule in post-transcriptional gene regulation. The complexity of gene regulation includes not only genes and mRNAs but also miRNAs [9]. In gene regulation mechanisms, the first and primary mechanism is mRNA cleavage which initiates translational repression and mRNA degradation processes [10]. RNA binding proteins (RBPs) play an important role in a process called miRNA-mediated translational repression by associating with argonaute family protein (Ago) directly or indirectly [11, 12]. Ago protein regulates mRNA target degradation through endonucleolytic cleavage. This pathway is also influenced by another process in which RBPs bind to various mRNA protein complexes. Exonucleoside degradation and restricted translational initiation also regulate mRNA expression [13].

## Processing and transcriptional regulation of miRNA

Before becoming a functional miRNA complex, miRNAs must go through several steps of processing [14]. miRNA processing is divided into canonical and non-canonical pathways. The canonical pathway engaged four chronological sequences in miRNA transcriptional regulation. The miRNA gene is first converted into pri-miRNA by RNA polymerase II, then the microprocessor complex converts pri-miRNA into precursor miRNA (pre-miRNA) further, the exportin 5 (EXP5/XP05) protein transports pre-miRNA from the nucleus to the cytoplasm, and finally the dicer enzyme aids in the formation of mature miRNA from the pre-miRNA [15]. The canonical pathway begins with the formation of the pri-miRNA transcript [16, 17]. Once miRNA is bound to the RNA-induced silencing complex (RISC), RNA polymerase II/III participates in this process post-transcriptionally or co-transcriptionally [18, 19]. The stem loop is dissociated off by the microprocessor machinery, Drosha complex, to constitute 60–100 nucleotide extended pre-miRNA that is further processed into 22 nucleotide long mature miRNAs by Dicer, an RNase III enzyme [20]. After that, pre-miRNA is exported to mature miRNA, duplex formation occurs in the cytoplasm with the help of exportin5/RanGTp. The Ago family proteins are loaded either 5′ or 3′strend on mature miRNA duplexes by a miRNA-induced silencing complex (miRISC) arrangement [21, 22]. In non-canonical pathway miRNA biogenesis is divided into two categories: Drosha/DGCR8-independent and Dicer independent pathways. Different protein combinations, such as Drosha, Dicer, exportin, and Ago2, are associated with the non-canonical pathway. In the absence of the Drosha/Dgcr8 complex, pre-miRNAs are processed in the nucleus. The Dicer enzyme is used for miRNA biogenesis in Drosha/DGCR8-independent pathway. Whereas miRNAs are cleaved in the presence of Drosha in the Dicer-independent pathway. These pre-miRNAs are associated with Ago2 for maturation after cleavage [23, 24]. After cleavage miRNAs transported with the help of XPO complex. Thus, Drosha or Dgcr8 occupancy benefits the canonical pathway but prevents non-canonical miRNA biogenesis. After that, miRISC complex binds to target mRNA to inhibit translation in both canonical and non-canonical pathway [4]. It is estimated that by targeting miRNAs, one-third of human genes can be directly regulated. Thus, the unique arrangement of miRNAs in different cell types regulates thousands of mRNAs under specific conditions [9, 25].

## Post-transcriptional regulations and gene silencing

The term 'epigenetic' refers to reversible changes in gene inheritance. DNA methylation, histone modifications, and small ncmiRNAs all play a role in epigenetic gene regulation. Small ncmiRNAs exert control over targeted gene expression through targeted mRNA degradation and translational inhibition [26]. According to Piletic et al*.,* there is a strong link between miRNA dysregulation, epigenetic changes, and disease development. Almost half of the miRNA genes have been identified as having correspondence to cytosine-phosphate-guanine (CpG) islands, which are modulated by the DNA methylation mechanism. Furthermore, miRNA expression may differ in diseased conditions compared to normal physiological conditions, indicating that miRNA has a disease-specific methylation pattern. Hyper- and hypomethylation of miRNA promoters has a direct impact on disease pathophysiology and pathogenesis in a number of disorders, including neurological, cardiovascular, autoimmune, and cancer. As a result, there is an unmistakable link between epigenetic changes, miRNA gene regulation, and disease development [27].

## Pharmacoepigenetics

Pharmacoepigenetics and pharmacoepigenomics are current epigenetic variation-based studies that describe the role of epigenetic mechanisms in regulating drug absorption, distribution, metabolism, and excretion (ADME) in terms of gene expression. miRNAs influence gene expression and ADME properties. miRNA contributes post-transcriptionally by binding to the 3′-untranslational regions (3′UTRs) of mRNA, influencing the performance of cellular processes and drug response at the genetic level [28–30].

### Drug metabolic cascade regulation of miRNA

The drug metabolic cascade is primarily divided into three phases in epigenetic pharmacotherapy. Changes in enzyme catalysed reactions such as oxidation, reduction, and hydrolysis are included in Phase I. Biotransformation or conjugation reactions are included in Phase II. Phase III includes drug uptake and elimination via membrane transporter proteins [31]. These drug metabolic phases govern a drug's efficacy and affinity. A genetic change may alter drug response and metabolic mechanisms [32]. Given the changes in drug ADME properties, methylation at the CpG promoter region, acetylation at the histone region, and miRNA mutation may all influence gene expression at the post-transcriptional level. Subordinating miRNAs influences epigenetic remodelling, drug metabolism, and drug response [33]. miRNA plays an important role in drug response by participating in the drug-metabolizing process and influencing the cytochrome P450 family (CYP) enzymes [34].

### Endogenous and xenobiotics (drugs) regulations of miRNA

Understanding drug efficacy and variability requires knowledge of drug-metabolizing enzymes (DMEs) and drug transporters (DTs). It is discovered that miRNA has an impact on post-transcriptional gene regulation in DMEs and DT. miRNAs may play a role in drug absorption, metabolism, and deposition [33]. Several miRNAs play critical roles in driving drug-metabolizing gene expression. In a carcinoma cell line, miRNA27-b and miRNA-378 suppress the expression of cytochrome P450 (CYP) cascade enzymes, CYP1B1 and CYP2E [35]. miRNA27-b and mmu-miR-298 also influence CYP3A4 expression. The CYP7A1 mechanisms are directed by miRNA-122a and miRNA-42a. miRNA-125 and miRNA-126 regulate the enzymatic mechanisms of CYP24A1 and CYP2A3 [36]. miRNAs also regulate the activity of drug transporters such as ATP binding cassette (ABC) and solute carrier (SLC) transporters. These transporters are used to control the absorption, distribution, and elimination of drugs [37]. ATP-binding cassette, subfamily B, member 1 (ABCB1/MDR1/P-gp) capable of transporting abundantly synthesised and naturally occurring molecules. It may also serve as a vehicle for chemotherapeutic drugs, steroids, different dyes, and peptides [38]. There are numerous miRNAs that are responsible for ABCB1/MDR1/P-gp variation such as miR-331-5p and miR-451 by direct regulation and miR-21 and miR-125b by indirect regulation [39]. miRNA up-regulation and down-regulation can cause changes in mRNA expression, which can lead to drug resistance in a variety of diseases. According to the research, miRNA-451, miRNA-27a, and miRNA-3315p target ABCB1 mRNA, resulting in negative regulation and drug resistance in various cancer cell lines. ABCB9 transporter expression is known to be regulated by miRNA-31. miRNA-326, miRNA-1291, and miRNA-134 are associated with ABCC1 effect modulation and are involved in drug resistance. The interference of miRNA-379, miRNA-9, and miRNA-128 in various cancer malignancies reduced the expression of ABCC2, ABCC3, and ABCC6, as well as the appearance of ABCC4, ABCC5 targets. Furthermore, drug resistance was observed in ABCG2 transporter modulation with interrelationships through several miRNAs such as miRNA-519c, miRNA-520h, miRNA-328, miRNA-212, miRNA-181a, and miRNA-487a in various cancer conditions [36, 40] (Table 1).

**Table 1:** The association of miRNA with the drug metabolising cascade

miRNA	Drug metabolizing cascade	References
miRNA 27-b, miRNA 378	CYP1B1andCYP2E1	[35]
miRNA 122a, miRNA422a	CYP7A1	[210]
miRNA 125, miRNA 126	CYP24A1, CYP2A3	[211, 212]
miRNA-451, miRNA 27a miRNA-3315p	ABCB1	[39, 213]
miRNA 31	ABCB9	[214]
miRNA 326, miRNA 1291 and miRNA 134	ABCC1	[215, 216]
miRNA 379, miRNA 9 miRNA 128	ABCC2, ABCC3 and ABCC6, ABCC4, ABCC5	[217–219]
miRNA 519c, miRNA 520h, miRNA 328, miRNA 212, miRNA 181a miRNA 487a	ABCG2	[40]

## miRNA-based therapeutic strategy

According to human genome research, RNA transcripts outnumber protein-coding genes. This significant finding challenges the central dogma that one RNA produces one protein. Non-protein-coding RNAs (ncRNAs) such as miRNA, natural antisense transcripts, piwi-interacting RNA (piRNAs), and long non-protein-coding RNAs (lncRNAs) are being developed as therapeutics [41]. The ability of miRNAs to drive the expression of billions of genes demonstrates the multi-targeting capacity of miRNA therapeutics. miRNAs have evolved as a prognostic biomarker for disease prediction, such as cancer, viral infection [42], neurodegenerative disease [43, 44], cardiovascular disorder [45, 46], diabetes [47] and muscular disorders [48].

### miRNA inhibition

miRNA therapeutics are thought to be divided into two categories: miRNA inhibition and miRNA replacement [49]. miRNA inhibition is a technique that employs synthetic, chemically modified single-stranded antisense oligonucleotides that are complementary to the 3′ end of a mature miRNA. It regulates miRNA action by inhibiting and suppressing the disease mechanism. Locked nucleic acids (LNA), chemically modified antago-miRNAs, phosphorodiamidate morpholino oligonucleotides (PMOs), and peptide nucleic acids (PNA) are examples of anti-miRNA oligonucleotides (AMOs). miRNA sponges or miRNA masking, a CRISPR/Cas9-based genome editing technique that modifies the genome of cancer cells, and small molecule miRNA inhibitors can also be used as miRNA therapeutics [50].

#### miRNA inhibition through anti-miRNA oligonucleotide (AMOs) or antisense-oligonucleotides (ASOs)

This method was used successfully for multiple miRNA targeting. LNA has a bi-cyclic structure with a locked furanose ring, and it serves as a platform for other analogues to improve binding capacity [51, 52]. The LNA structure is built around mixmers and gapmers. In mixmers, LNA and DNA nucleosides are dispersed throughout the oligonucleotide sequence, whereas gapmers have two LNA fragments separated by a DNA/Phosphorothioate (PS) nucleoside gap at both ends of the oligonucleotide. Because gapmers have DNA/PS linkages, they recruit the RNA-cleaving enzyme RNase H and fascinate to downregulate mRNA expression, resulting in decreased protein translation [53, 54]. Single-strand antisense molecules are used in this mechanism to diversify mature miRNA activity via remodelling approaches. The methylene bond between the 2′-O and 4′-C of ribose in the LNA structure improves LNA stability and hybridization [55]. Various modifiers, such as 2-O-me, 2′F, 2′NH2, 2′H, phosphorothioates, and locked nucleic acids, combine with the miRNA mimic to increase its half-life. Modifications to protein binding characteristics can be in the form of sugar bases, nucleotide, or inter-nucleotide bond transformation. This modifier group stimulates miRNA hybridization, inhibition, target specification, endonuclease resistance, and seed strand melting temperature. These types of oligonucleotide corrections increase binding capacity, nuclease resistance, the delivery system, and support endocytic transport [56, 57]. Chemical modification improves pharmacokinetic properties, and phosphorothioate backbone modification protects oligonucleotides from degradation and increasing binding affinity with plasma proteins. This reformation, like 2′-methoxy or methoxyethylene modifications, increases the stability of oligonucleotides at lower doses [58]. AntagomiRNAs primarily target oncomiRNAs in order to develop potential treatments for oncogenesis as well as other diseases [59]. Several in vivo and in vitro experiments confirm miRNA-based inhibition for the establishment of chemical modification-based drug delivery. HeLa cells were the first to be used in the study of sequence-specific inhibition of miRNA activity using 2′-O methyl (2′-O-Me) modified RNA oligonucleotides paired with mature miRNAs. Using antimiRNA oligonucleotides 3′ cholesterol-conjugated antagomiRNAs, miRNA silencing has been reported to be successful in a variety of animal disease models [50]. A 2′-O Me group-modified oligonucleotide has been identified as a potent inhibitor in a variety of cancer cell lines [60]. Some ASOs are in clinical trials, such as MRX34, a synthetic double-stranded RNA oligonucleotide that works on miRNA-34 and repairs its mechanism on the p53/wnt cellular pathways. Chemically modified nucleotides with phosphorothioate linkage, cholesterol-conjugated single-stranded RNA analogues antagomirs' paired with miRNA-122 strand significantly regulate liver disease mechanisms and can function as a miRNA-based inhibitory therapeutic target for antiviral approach destruction [61]. Peter Sarnow coined the phrase "miRNA-122 targets the 5′ non-coding region (NCR) of the HCV genome and up-regulates viral RNA activity" in 2005. Numerous studies have confirmed the role of miRNA 122 in liver diseases. Miravirsen (also known as SPC3649), an LNA-based antisense molecule against miR-122 developed by Santaris Pharma A/S (Horsholm, Denmark) in Phase 1 and Phase 2a clinical trials for the treatment of hepatitis C (HCV) [62]. The LNA-mediated drug cobomarsen (also known as MRG-106) is being tested in clinical trials for the treatment of cutaneous T-cell lymphoma (CTCL), diffuse large B-cell lymphoma, chronic lymphocytic leukaemia, and mycosis fungoides (MF) [63, 64]. In another xenograft murine model-based metastatic breast cancer study, blocking of miRNA 10b activity via intravenous delivery of specific antagomiRNAs leads to a significant reduction in the development of lung metastasis [65]. Phosphorodiamidate morpholino oligonucleotides (PMOs) are single-stranded DNA oligonucleotides derived from AMO that have additional morpholine rings linked with phosphorodiamidate linkages. PMOs inhibit the mRNA cascade by coupling with the corresponding sequence of target mRNA via Watson and Crick base conformation. PMOs are stable oligonucleotides that are susceptible to a variety of biological enzymes, increasing their therapeutic potential. The US Food and Drug Administration has now approved EXONDYS 51TM (Eteplirsen), a PMO-based antidote that works in Duchenne Muscular Dystrophy (DMD). This drug works on a mutated dystrophin protein to repair its function by removing exon 51 via RNA splicing [66]. Peptide Nucleic Acids (PNA) are composed of N-2-aminoethyl glycine components that have been conjugated with peptide bonds. PNAs nucleotide corresponds to recognise complementary sequences in a specific gene by the fascinating transcriptional process in the antigene approach [67]. PNAs with the ability to form a stable triplex structure, as well as a strand invasion or strand displacement complex with DNA. These structures have the ability to disrupt or chunk RNA polymerase activity. Antisense nucleic acid analogues are possibly designed for identifying mRNA sequences for translation inhibition. PNA-dependent antisense therapy works by sterically inhibiting either the RNA mechanism or translation. Antisense delivery systems are being developed [68]. Formiversen is the first antisense-based drug approved to treat cytomegalovirus retinitis (CMV) in AIDS patients. Several antisense-based treatments are currently being tested in clinical trials. For example, Geasense, an antitumour activity antisense drug with active Genta targeting BCL 2, is in phase III trials [53, 69]. PNAs, when compared to other oligonucleotide therapeutics, are ineffective at entering the cell membrane and have low intracellular delivery due to their neutral charge [70]. AMOs-based delivery systems for targeting mature *miRNA* have proven to be effective. Chemical modification of oligonucleotides provides a more powerful tool for AMOs-based target identification in miRNA biology (Table 2).

**Table 2:** Approved miRNA-based drugs for miRNA therapeutics

Drug	Clinical trial	miRNA inhibition	Disease	References
MRX34	Phase I	ASOs (2-O′ methyl modifier)	P53/wnt pathway	[61]
Miravirsen (SPC3649)	Phase I and Phase IIa	phosphorothioate linkage, cholesterol-conjugated AMOs	HCV	[62]
Cobomarsen (MRG-106)	Phase II	LNA based	Various lymphomas	[63]
Formiversen	Phase III	PNA based	CMV	[220]
EXONDYS 51TM	Approved drug	PMO based	Duchenne muscular dystrophy (DMD)	[66]
Geasense	Phase III	PNA based	BCL2	[53, 69]

#### miRNA inhibition through miRNA sponges/miRNA masking

miRNA sponges (miR-SP) are another vector-based concept with 4–10 multiple binding sites complementary to the seed area of the target miRNA [71, 72]. miRNA sponges are linked to the transcriptome category, which has been identified as competing for endogenous RNA (ceRNA). This ceRNA serves as a socking up and controlling miRNA expression by directing the RISC capacity and decreasing mRNA target efficiency [73, 74]. Chemically modified antisense oligonucleotides have the same inhibitory ability as miRNA sponges [75]. Sponge types in the miRNA sponge-based inhibition mechanism include target mimics, miRNA decoys, miRNA target sequences, miRNA erasers, lentiviral-mediated antagomiRNAs, and non-viral delivery systems [71]. miRNAsong is a computational tool that has been developed to predict miRNA sponges. miRNA sponges are created for specific miRNAs, and binding potentialities for specific organisms have been identified. This web-based programme contains 35,528 miRNA sequence collections that serve as the foundation for sponge structure construction [76]. miRNA masking, like miRNA sponges, is another avenue for miRNA-based inhibition. Unfavourable miRNAs block their target mRNA in the masking technique, and thus, target gene expression may be inhibited. This masking strategy employs a gene-specific method of binding with miRNA targets in a perfectly complementary mode [77]. Masking and sponge strategies collaborate to further inhibit multiple miRNA binding sites, resulting in protein synthesis suppression. Sponge miRNA masking is moderately coupled with target complementary, so this combination has low gene target selectivity, but it may be advantageous for targeting all genes that are linked to the same binding lineage of miRNA group [78–80]. Using a viral-based delivery approach, the systemic application of miRNA sponges has shown great promise in the eye [81], skeletal muscle [82], in vivo study model for heart disease [83] and carcinogenesis [84]. Meng et al*.,* summed it up as sponge's block. miRNA-9 expression is generally responsible for regulating or suppressing various oncogenes such as KLF17, CDH1, and LASS2 using circular single-stranded DNA (cssDNA) by binding with miRNA-9 target sites and helping to repair the expression of these oncogenes [85]. A liposome-based miRNA sponge mimic is currently being tested in a phase I trial for primary liver tumours [45]. These miRNA sponges or decay prospects could also be useful in regenerative medicine [86]. Using an ex vivo model, lentiviral particles were designed for bone marrow cells to deliver anti-miRNA sponge target sequences for miRNA-144 and miRNA-451. These findings indicate that lentiviral coded anti-miRNA studies can be carried out successfully [87]. Another animal model for autoimmune encephalomyelitis (EAE) in mice shows decreased activity of interleukin-17 secreted by Th-17 cells by introducing a lentiviral sponge for miRNA-326 [88]. The concept of miRNA sponges is also useful for chronic disease prevention, such as diabetes [89]. Another method for generating a maximum removal of endogenous miRNA is to use a miRNA eraser. miRNA erasers have two binding sites and are delivered to cells via recombinant adenovirus. This miRNA eraser mechanism is useful for engineered transgenic models and may be useful for in vivo therapeutic miRNA targeting [90]. It is critical for the translation of miRNA-based inhibition sponges, erasers, masking decoys to have an effective design model but be inconclusive about its safety, efficacy, and off-target effects [91]. Nonetheless, sponge-based inhibition is a compelling approach, but clinical translation of this concept will be difficult. Furthermore, the toxicity of nanoparticles liposome-based therapy, specified delivery in tissue, low cellular uptake, uncertainty in animal-based experiments, control vector system, and the toxicity of nanoparticles liposome-based therapy are major concerns [86].

#### miRNA inhibition through CRISPR/Cas9-based genome editing

Genome editing is now widely accepted as a molecular technique for modifying genes and their expression. Clustered regularly interspaced short palindromic repeats (CRISPRs) and CRISPR-associated protein 9 (Cas9) are potential gene targeting technologies that allow modifications at the DNA level with precise chromosomal locations in cells and animal models [92]. CRISPR/Cas elements have an adaptive antivirus immunity system, which is found primarily in archaea and bacteria. This system operates on the basis of self and foreign recognition. Spacers are foreign DNA fragments that fit into the CRISPR compartment. This construct transcribed and proceeded with the formation of CRISPR RNA (crRNA), which can easily target and cleave genomic sites associated with plasmids or viruses [93]. The CRISPR/Cas mechanism is divided into three stages: spacer acquisition, crRNA biogenesis, and target interference [94]. There are three types of unique feature genes in CRISPR-Cas: Cas-3 in the type I system, Cas-9 in the type II system, and Cas-10 in the type III system [95]. The CRISPR-Cas9 system influences tumour hallmarks such as cell growth and proliferation, migration, invasion, and apoptosis in cancers such as hepatocellular carcinoma, renal cell carcinoma, pancreatic cancer, ovarian tumour, and chronic myeloid leukaemia. In vivo and in vitro experiments yielded significant results for a variety of targeted miRNAs and their regulating pathways [96]. Lu et al. conducted a CRISPR-cas9-based clinical trial, and CRISPR-Cas9 modified T cells were recently introduced to lung cancer patients for treatment purposes [97, 98]. Furthermore, CRISPR-Cas9 tools are applicable for HIV-1 strains, as CISPR-Cas9 acts on both HIV-1 and proviral DNA in in vivo and in vitro models. The CRISPR-Cas9 method inhibits HIV viral gene expression and viral replication. This technique is simple, has a high efficiency, and has a low off-target effect, making it a potential and hopeful treatment for HIV-1/AIDS [99]. Currently, miRNA-based inhibition with LNA/PNA modifications, blocking by miRNA sponges, and knockdown approaches through genomic editing are expanding research in the drug delivery system [100] and CRISPR-Cas may be useful as a therapeutic platform for gene therapy [101, 102]. Despite its powerful genome-editing system, there are numerous gaps, such as success, safe, and efficient target distribution in human subjects. [103].

#### miRNA inhibition through small molecule inhibitors of miRNAs

Small-molecule inhibitors of miRNAs (SMIRs) are emerging as nucleotide analogues to target miRNA and modulate their activity in the development of miRNA therapeutics [9]. Melo and Calin et al*.* pioneered the use of SMIRs [104, 105]. SMIRs are a potential option that could be an effective concept for the direct inhibition of disease-associated miRNAs by binding with a precursor or mature miRNA [105]. AC1MMUR2 inhibits the maturation of pre-miRNA-21 in the in vivo mouse model, resulting in tumour suppression [106]. Gumireddy et al*.* strained over 1000 organic compounds and discovered that diazobenzene and its derivatives precisely inhibit pre-miRNA-21 transcriptional activity by interfering with the miRNA pathway's subsequent process [107]. SMIRs with promising pharmacodynamic and pharmacokinetic properties are followed by a concise clinical application regarding their toxicity and distribution. Because of their cost-effectiveness, SMIRs stand out as potential candidates for drug targeting due to their intriguing output [75, 100].

### miRNA replacement

miRNA replacement therapy is gaining traction around the world. Synthetic miRNA or miRNA mimics are incorporated with diseased tissue/cells to restore normal functions such as cell proliferation, cell apoptosis, cell cycle, and other cellular and physiological activity [108]. miRNA replacement therapy is classified as either viral or non-viral delivery [109].

#### Viral vector-based miRNA replacement

Retroviral, lentiviral, and adeno-associated viral (AAV) vectors are used in viral vector-based transportation for miRNA administration, which encodes an RNA molecule and can transfect all cell types because it affects both dividing and non-dividing cells. Because of its low toxicity and limited off-target delivery, an adeno-associated viral vector has an idealistic effect [110, 111]. AAV vectors with genomic sizes of up to 4.7 kb were found to be shorter than other viral vectors. It aimed at both dividing and non-dividing cells in order to deliver viral vectors [112]. AAV also has a protein capsid with single-stranded DNA [113]. In an animal model of miRNA replacement therapy, Kota et al*.,* discovered that miRNA-26a combines with AAV and is introduced into hepatocellular cancerous cells. They discovered that increased miRNA-26a expression leads to a significant reduction in tumour growth and induced cell apoptosis. This demonstrates a potential targeting technique for future applications [114]. Retroviruses have a lipid layer that contains two copies of a linear non-segmented single-strand RNA molecule that is transcribed into double-stranded DNA in the cytoplasm before being passed into the nucleus and assimilated with host chromosomes [115–117]. Retroviruses have a genomic size of 7–11 kb and the ability to construct vectors of up to 8 kb for interest gene transfer. These viruses only affect dividing cells because they affect the mitotic phase of the cell cycle. In comparison with retroviruses, lentiviruses can create a space for genomic transfer of up to 8 kb but can affect both differentiated and non-differentiated cells. Lentiviruses are a subgroup of retroviruses that have a protein-protecting shell and the ability to duplicate a copy of their single-stranded RNA genome [118, 119]. A recent animal model-based study concluded that the lentiviral vector was designed with miRNA-133b for the restoration of spinal cord function [120]. Despite being favoured as an excellent therapeutic representative, viral-based delivery failed due to low loading capacity, high toxicity, limited packing ability, difficulties in viral and vector production, and extreme immunogenicity [103]. Non-viral vector-based delivery systems have been elevated to overcome all of these challenges (Table 3).

**Table 3:** Involvement of vectors in miRNA-based delivery

Vector	Size	Structure	Influence on cells
AAV	~ 4.7 kb	Protein capsid with ss DNA	Dividing and non-dividing cells
Retroviruses	~ 7 to 11 kb	Lipid layer ss RNA	Only dividing cells
Lentiviruses	~ 8 kb	Protein capsid ss RNA	Dividing and non-dividing cells

#### Non-viral vector-based miRNA replacement

There are two types of delivery approaches in non-viral therapeutics. Oligonucleotide-based miRNA mimics are delivered chemically via liposomes, nanoparticles, conjugation-based, and antibody-based methods. miRNA are delivered by gene gun, electroporation, hydrodynamic, ultrasound, and laser-based energy in the physical approach [121]. Because of its membrane-like structure and capsulated cover, lipid or nucleic acid is frequently used as a carrier for miRNA in liposome-dependent therapy. Liposomes are classified as cationic, anionic, or neutral lipids based on their electrical charge [122]. Cationic charged lipid complexes have a higher absorption capacity because electrostatic interactions effectively adhere to anionic plasma membranes [123]. Nanoparticles (NPs) can be used as a carrier for miRNA in the chemical-based concept for miRNA delivery. NP degrade slowly by endonucleases and avoid non-specific binding with other protein targets [124]. miRNA delivery via lipids, polymers, and inorganic nanoparticles has been used [125]. Lipid conjugated dsRNAs containing palmitic acid, lauric acid, and cholesterol are used in the delivery of nonviral miRNAs via conjugation. These lipids have been modified at the 5′ end of the sense strand in order to improve cellular uptake and add efficacious miRNA delivery [126]. For the development of miRNA-based therapeutics, conjugation linked targeting employs peptides, antibodies, and bioactive molecules. In the antibody-dependent approach, the antibody-based targeting carrier was shown to be effective for both systemic and cell-specific delivery of oligonucleotides by targeting cell surface receptors [127].

The physical method of gene transfer is used in gene therapy in a variety of cell types both in vitro and in vivo. These methods rely on causing transient dispersion in the cell membrane by applying mechanical, electrical, ultrasonic, hydrodynamic, or laser-based energy to the desired DNA as it diffuses into host cells [128]. The gene gun is a physical device used to inject the DNA particle. Plasmid DNA is coated with gold and tungsten particles before being delivered to host or target cells using a high-speed pressurised gas. This biolistic technique, which was originally developed for plant transgenesis, is now used in mammalian cell gene delivery in the in vitro and in vivo settings.

One of the most widely used gene delivery methods is electroporation. Skin, skeletal muscle, liver, tumour tissues, retina, brain, spinal cord, kidney, cardiac muscle, and cornea were successfully transfected in vivo using the electroporation technique [129]. miRNAs were packed into extracellular vesicles and electroporated into target cells for successful gene delivery [130]. UMTD (ultrasound-targeted microbubble distraction) is a non-invasive method for miRNA and gene delivery. Yanlei et al*.* investigated the effect of UMTD on miRNA-133a for breast carcinoma treatment in MCF-7, MDA-MB-237, as well as on in vivo model and reported that miRNA-133a suppressed cell proliferation in vivo model tumour growth has been suppressed significantly at low frequency (10 MHz) [131]. In another study, Jennier et al*.* suggest that by delivering UMTD miRNA-122 loaded nanoparticles, the concentration of interleukin-12 and 17 on lymph nodes of treated and contralateral tumours in hepatocellular carcinoma was reduced [132]. Another study looked at UTMD-mediated miRNA delivery in cancer stem cells and found that it could be a platform for cancer stem cell (CSC) therapy [133]. Gene delivery systems have been widely used in a variety of diseases, including ocular diseases [134], cardiovascular dysfunction [135], neurodegenerative diseases [136] and inflammatory disorders [137]. Magnetofection is another method for delivering miRNA. The molecules were delivered into the target cells using magnetic fields. The magnetofection method efficiently examines all types of oligonucleotides, DNA, RNA, and viruses in a wide range of cell lines. According to one of the studies, magnetic nanoparticle neuromeg was discovered to be used to transport oligonucleotides for miRNA inhibition via magnetofection. This neuromeg complex is capable of successfully knocking down miRNA-134 expression in the rat brain region, as well as reducing the content of overexpressed specific miRNAs for brain disorders. [138]. Hydrodynamic pressure aids in the formation of pores in the hydrodynamic strategy, allowing genes of interest to enter the cell through these pores [139].

**Fig. 2:**
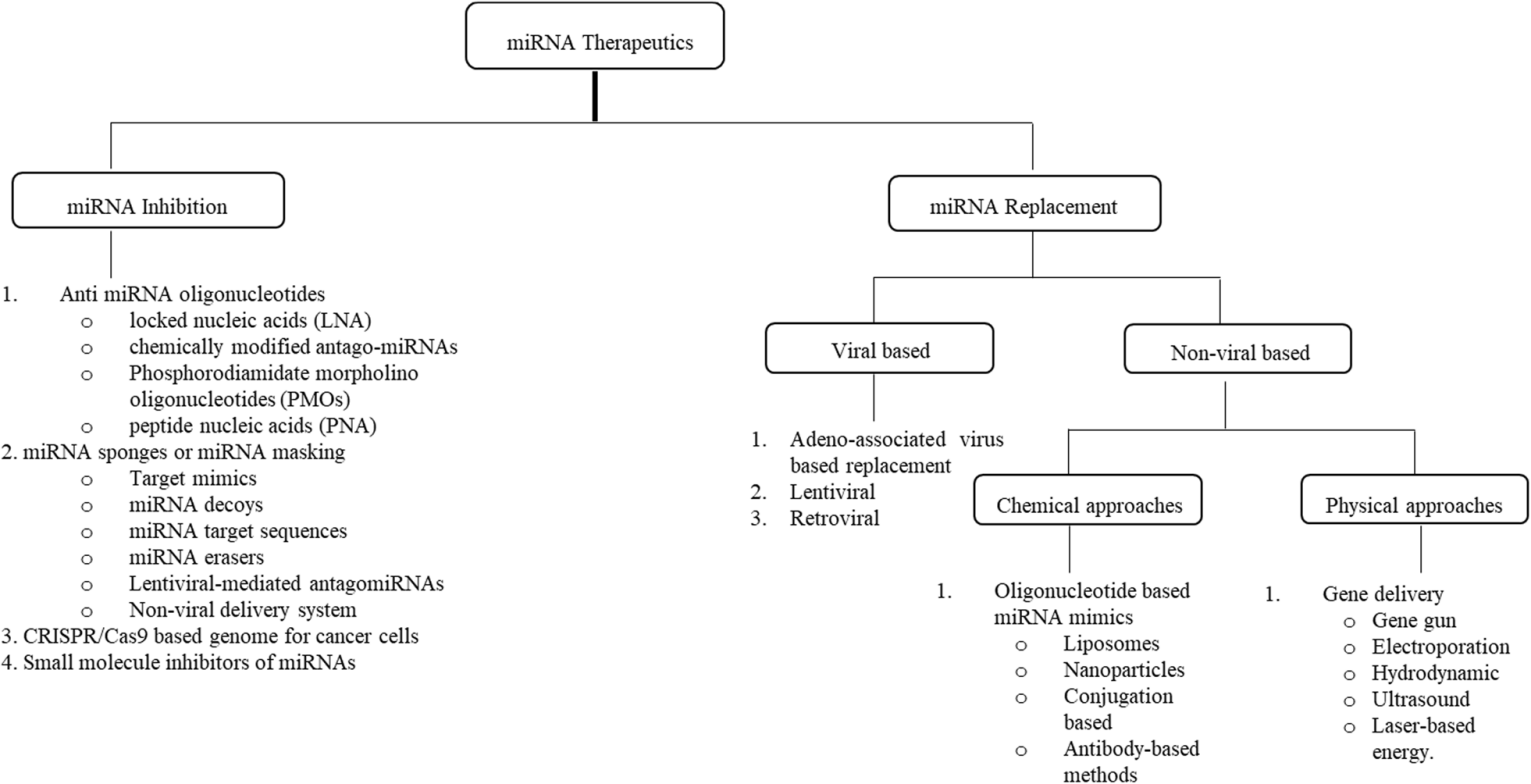
The diagram depicts the divisions of miRNA-based delivery therapies

Hence, miRNAs play an important role in therapeutics and may become a future medicine. Currently, small RNA-based therapeutics are a hotbed of innovation and an important area for obtaining patent rights. The study discovered 87,700 patents related to miRNA using "Google patent". Another quick search of US and European patent databases revealed that the number of patents related to "miRNA and cancer" was disproportionate to other diseases [140]. Traditional and complementary medicines have been globally accepted as an alternative form of therapy for various chronic disorders and diseases, according to the WHO Traditional Medicine Strategy 2014–2023 [141] (Fig. 2).

## Plant miRNA in therapeutics and its biogenesis

Plant miRNAs have 22 nucleotide RNAs. Because of the lower diversity in linage classification, the biogenesis and mechanism of these plant miRNAs show less discrimination when compared to animal miRNAs. Plant miRNA differs from animal miRNA only in terms of miRNA stem-loops; plant-predicted fold backs are larger than animal miRNAs [28]. According to Axtell and Meyers, plant miRNA are derived from distinct stem regions containing single-stranded hairpin precursors. It covers all biological and physiological processes. It also regulates environmental stress [142]. The plant genome transcribed a large number of miRNA genes [143, 144]. The RNA polymerase II enzyme is responsible for the transcription of primary miRNAs (pri-miRNAs) in plants. In the nucleus, DICER-LIKE 1 (DLC1) converts pri-miRNAs into stem-loop pre-miRNAs. These pre-miRNAs were transported into the cytoplasm by the plant exportin 5 orthologue, HASTY, and other unknown factors. Mature miRNAs are formed from the strands of pre-miRNAs and methylated by a small RNA methyltransferase called HUA Enhancer 1 (HEN1) [145]. The response to miRNA silencing is carried out by AGO-containing RNA induce silencing complex proteins that work on the guide miRNA strand [42, 146, 147]. Animal miRNAs lack a 2′O-methyl group on their 3′-terminus, which is found in plant miRNAs. miRNA forms near-ideal or ideal base-pair complementarity for target identification in plants. According to the 'seed rule,' animal miRNAs lack this complementarity [145]. miRNAs play a role in plant development, plasticity, abiotic/biotic responses, and symbiotic/parasitic interactions in plant-environment relationships [148].

## Application of plant miRNA in cross-kingdom regulation

A wide range of bioactive compounds have pharmacological functions that can be classified by screening various phytochemicals [149]. Medicinal plants and their secondary metabolites are used as remedies in the form of foods and pharmaceuticals. Secondary bioactive metabolites such as polyphenols, alkaloids, saponins, and tannins are important in drug therapeutics. Although the synthetic medicinal plant variants are not same as the complex plant components [150]. Plant miRNAs influence biosynthesis processes and have emerged as therapeutic markers for a variety of diseases. A novel and contentious hypothesis suggests that miRNAs can be transferred from one species to another and can potentially regulate target genes in distant species [151]. When it comes to miRNA transfer from plants to humans or animals, the situation becomes more convoluted. This is due to a number of unanswered questions and contradictory findings concerning plant miRNA stability, abundance, mode of action, and validation of potential targets in human cells [152–154].

## Bioinformatics validations of plant miRNA in trans-kingdom systems

Plant miRNA is involved in a variety of biological processes. It not only regulates plant processes, but it also has a noticeable effect on the activity of other species. This suggests that plant miRNAs play a role in inter-species regulation. There are numerous bioinformatics tools and software available for the anticipation of bioinformatics studies such as plant miRNA design, implementation, validation, target prediction, line of conduct within the mammalian host cell environment, and understanding complementarity between cross-kingdom interactions [155, 156]. There are four main characteristics that are commonly used when designing herb-based miRNA for mammalian genes. Base-pairing between the 'seed' region and the target gene, low free energy estimation (genuine paring with miRNA target), target prediction (potential binding sites required for cross-kingdom transfer), and site accessibility are examples of these. For the prediction of plant-based miRNA transfer in mammalian genes, these rules are well-founded [157].

There are several bioinformatics databases for plant and animal miRNA prediction, and miRNEST2.0 is one of the comparative tools used for plant and animal miRNA. This application recognises, develops, and integrates miRNA data. It also incorporates miRNA sequences from other databases and prepares miRNA annotation for the selected species [158]. Another computational tool for identifying plant miRNA sequences and biological precursors is miRBase. It also provides information about genome coordinates and context, as well as references to the literature, deep sequencing expression data, and community-driven annotation [159]. MiRanda [160], TargetScan [161], PITA [162], PicTar [163] and COMIR [164] are tools for miRNA-mRNA interactions. miRU is a well-known tool for analysing plant miRNA targets [165]. psRNATarget is a tool for determining the reverse complementarity between small RNA and target transcript and target-site. It also assesses target accessibility by computing unpaired energy (UPE) [166].

### In-silico predictions for the natural compound in cross-kingdom pass-over

Maulik et al*.* conducted a recent study on *O. basilicum* miRNA concerning plant miRNA pass-over in the human system and in silico investigation. The study identifies eight conclusive miRNAs and 87 predicted target genes that are linked to a regulatory pathway. It has cross-kingdom effects on the RAS/MAPK signalling cascade, cardiomyopathy, HIV, breast cancer, lung cancer, Alzheimer's, and several neurological disorders [167]. The computational characterization of *Bacopa monnieri* revealed that it performs effective regulatory tasks. In this study, 12 *B. monnieri* miRNAs were found to have physiological functions and target 68 human genes, indicating certain transducing cascades such as NF-kB and MAPK, revealing miRNA-mediated cross-kingdom gene regulation [168]. Furthermore, *Gmelina arborea* miRNA cross-kingdom transfer appears to be a key regulator of a number of human abnormalities. It is effective for stomach, liver, gynaecological disorders, fevers, and skin problems. There are six accepted miRNAs and 73 *G. arborea* associated genes. Bioinformatics analysis has become accustomed to influencing human processes such as signal transduction and apoptosis [169]. A comparable in vitro study confirms that *G. arborea* bark and fruit extract have antioxidative properties on liver culture cells by downregulating oxidative-induced damage in liver cells [170]. Other studies on *Moringa oleifera* show that its miRNAs have the potential to target a variety of human genes. It also stated that its miRNAs have anti-oxidative and anti-tumour activity [171, 172]. With cross-kingdom target prediction, eight presumed plant miRNAs of *M. oleifera* were identified through in silico investigation. *M. oleifera* miRNA-168a regulates the SIRT1 gene, which works in tandem with the p53 gene to regulate metabolism, stress signalling, cell survival, cell cycle control, and genome stability. Furthermore, a synthetic mimic of *M. oleifera* miRNA-168a was transfected in the human hepatoma cell line G2 (HEPG2), and its pharmacological properties need to be investigated for a better understanding of disease regulation [173, 174]. The anti-cancer effects were attributed to the *Viscum album, L.* Several in vivo* and *in vitro studies have shown that *V. album* extracts have potent cardioprotective [175], hypoglycemic and antihypertensive vasodilator effects [176]. A total of 14,559 target genes for 44 potential novel miRNAs were identified in the study of the *V. album* transcriptome. Using the bioinformatics engine, these miRNAs were identified as pharmacologically potent for targeting human genes and regulating pathways [177]. Noopur Singh et al*.* conducted a bioinformatics analysis on *Curcuma long*a and discovered that sixteen miRNAs from *Zingiber officinale* narrate gene modulation with 86 human targets. These target annotations are involved in a wide range of cellular and biological processes [178]. Another in silico study by Rashmi et al*.* discovered eight out of 12 miRNAs that play a role in gene silencing by stimulating various targets of signal transduction and apoptosis by admiring disease inhibition such as diabetes mellitus type 2, cardiovascular disorders, Alzheimer, cancer thalassemia. [179]. Similarly, *Camptotheca acuminate* is commonly referred to as a happy tree. Bioinformatics has been used to facilitate this plant's cross-kingdom association. Out of 53,294 *C. acuminate* miRNAs in the EST database, 33 highly stable predicted miRNAs were detected for human gene target associations, with 14 miRNAs identified to govern the 152 human target genes via prominent pathways such as focal adhesion, lipolysis regulation, and mTOR signalling. These pathways play an important role in cancer regulation. *C. acuminate* miRNAs may be an important clue for targeting cancer progression, but more research is needed [180]. In context to our findings, a total of 89 unique miRNA were discovered using miRbase against using expressed sequence tags (ESTs) in *Curcuma longa*. This shows direct target on anti-cancer and anti-immunosuppression activity. In our other study on miRNAs of *Persea americana* (Avocado), a total 243 putative miRNAs were disclosed and its predicted targets significantly involved in metabolic and cellular processes [181, 182]. Despite the fact that a large number of bioinformatics studies have been conducted to investigate the link between plant miRNA and trans-kingdom transfer, experimental validation and further in-depth research are recommended to fill the gap.

## miRNA therapeutics value in inter-kingdom regulation

Plants as a source of medicine have proven to be extremely useful in human life. They are used as a preventive medicine in treatment. There are approximately 252 drugs that play an important and fundamental role in human health, with plant-derived natural medicines accounting for 11% of the total. There is a wealth of information available about the chemically modified plant-based drug [183]. Nutritional elements such as vitamins and secondary plant metabolites are critical in driving the cellular mechanisms that maintain miRNA expression. Food-derived nutritional components such as vitamin D, vitamin E, folate, curcumin, resveratrol, epigallocatechin gallate, quercetin, and isothiocyanates have been recognised as miRNA moderators [184, 185]. Plant miRNAs regulate cross-kingdom gene expression, but the evidence for inter-species coordination is mixed [186]. Several recent discoveries show cross-kingdom gene efficiency by plant miRNAs in the model of cross-kingdom mechanism [187]. This aids in the investigation of the current disagreements surrounding this concept [188].

### Plant miRNA interactions: grassland to mammals

Plant miRNAs in the form of food can theoretically be absorbed by intestinal epithelial cells [160, 189]. Although the exact pathway of the plant's miRNAs from gut to intestinal cells is unknown. Several groups have demonstrated the potential mechanism and uptake of plant miRNA in mammals. According to Zhang et al*.,* diet-derived plant miRNA was found in the circulation and organs of humans and mice, and it was capable of regulating the expression of human mRNAs. Rice-derived miRNA-168a regulates gene expression effectively and has been shown to target low-density lipoprotein receptor adapter protein (LDLRAP) 1, a gene involved in cholesterol metabolism [190, 191]. Andrew et al*.* identified plant miRNA-159 as a dietary source in human sera and further recognised its potency to inhibit cell proliferation by pointing tcf7, which expressed wingless-related integration site (Wnt) signaling-associated transcription factor, prominent to a down-regulated myc gene regulation in breast cancer cells. Furthermore, in a mouse modal in vivo experiment, a mimic of miRNA-159 significantly inhibited xenograft breast cancer. Plant miRNA was found to be capable of controlling cancer growth in mammals in an in vitro experiment. [192]. Liu et al*.* describe how miRNA-2910 was discovered in human sera through the investigation of plasma sRNA sequencing datasets in the continuation of these cross-kingdom pieces of evidence. The data were removed from the miRBase domain, however, because it was an existing fragment of large subunit ribosomal RNA[193]. miRNA-172 from *Brassica oleracea* was found in the blood, spleen, liver, and kidney for up to 36 h, indicating the stability of miRNA, according to Liang et al*.* [194]. When human monocyte-derived dendritic cells were treated with fvmiRNA-168 and inflammatory agents such as lipopolysaccharide (LPS) or polyinosinic: polycytidylic (poly I: C) acid, a positive downregulation of inflammatory response was observed. Furthermore, fvmiRNA-168 could reduce class II immune-histocompatibility complex (HLA-DR), various CD markers, and T-cell proliferation [153]. Plant miRNA's medicinal properties will be prominent in the prevention of human maladies Wang et al*.* created a gene knockout mouse model that demonstrated that exogenous miRNA-451 can pass through circulation and the digestive system and up-regulate antioxidant activity via the Foxo3 pathway [195]. Plant miRNAs and their effects on cross-kingdom gene regulation provide evidence. Despite the unknown aspects of their delivery, potentiality, sustainability, toxicity, and function, researchers were faced with a number of challenges (Table 4).

**Table 4:** Plant miRNAs in gene regulation: a cross-species comparison

miRNA	Source	Year	Target of interest	Method	Disease application	References
miRNA-168a miRNA-156a miRNA-166a	*Oryza sativa*	2012	Human, Mouse, Rat, Calf, Hoarse, Sheep	HTS, qRT-PCR, NB, WB	Low-density lipoprotein receptor adaptor protein-1 (LDLRAP1)	[190]
miRNA1_GA_CONTIG1 miRNA2_GA_CONTIG1 miRNA3_GA_CONTIG1 miRNA4_GA_CONTIG1 miRNA5_GA_CONTIG1 miRNA6_GA_CONTIG1	*Gmelina arborea*	2013	Human genes	Bioinformatics analysis	Signal transduction and apoptosis regulation	[169]
08 predicted miRNA	*Curcuma longa*	2013	Human genes	Bioinformatics analysis	Diabetes mellitus type 2, cardiovascular disorders, alzheimer, cancer, thalassemia	[179]
miRNA-172	*Brassica oleracea*	2014	Mice	qRT-PCR	Not mentioned	[194]
miRNA-2911	Honeysuckle	2014	Mice	qRT-PCR, HTS, NB, florescent labelled tracing assay	Not mentioned	[204]
miRNA-29b, 200c	Milk derived	2014	Human, Mice	qRT-PCR	Not mentioned	[157]
miRNA-2911	*Lonicera japonica*	2015	Mice	qRT-PCR, HTS, NB, florescent labelled tracing assay	Influenza A virus	[121]
miRNA-375	Milk derived	2015	Mice	qRT-PCR, NB, HTS	Not mentioned	[221]
miRNA-168a	*Moringa oleifera*	2016	Human genes,	Bioinformatics analysis	Stress signalling, cell survival, cell growth, cell cycle and genome stability	[174]
miRNA-166a miRNA-159	*Brassica campestris*	2016	Mice	HTS, qRT-PCR	Not mentioned	[222]
miRNA-159	*Arabidopsis thaliana*	2016	Mice	qRT-PCR	Breast cancer/transcription factor 7	[192]
miRNA-159	Glycine max	2016	Mice	qRT-PCR	Breast cancer/transcription factor 7	[192]
miRNA-159	Broccoli	2016	Mice	qRT-PCR	Breast cancer/transcription factor 7	[192]
miRNA-14	*Curcuma longa*	2016	Human	Bioinformatics analysis	Rheumatoid arthritis	[178]
miRNA-160 miRNA-2673	*Brassica oleracea*	2016		qRT-PCR	Not mentioned	[223]
miRNA-2910	*Populous euphratica*	2017	Human	Bioinformatics analysis	JAK-STAT pathway	[193]
44 potential miRNA found	*Viscum album*	2017	Human genes	Bioinformatics analysis	Cancer, cardiovascular diseases and neurological disorders	[177]
14 potential miRNA	*Camptotheca acuminate*	2017	Human genes	Bioinformatics analysis	Focal adhesion, lipolysis regulation and mTOR signalling	[180]
miRNA-156a	Cabbage, spinach and lattuce	2018	Human	qRT-PCR	Cardiovascular diseases	[224]
miRNA-414 and miRNA-869.1	*Ocimam basilicum*	2019	Human genes	Bioinformatics analysis	Rheumatoid arthritis, Diabetes mellitus, Gestational diabetes, Cataract, Alzheimer’s disease, Infant death syndrome, Infantile achalasia and Cantu syndrome	[167]
Bmn-miRNA-167h Bmn-miRNA-168 Bmn-miRNA-396g Bmn-miRNA-156 Bmn-miRNA-172d Bmn-miRNA-171d-3p Bmn-miRNA-399h-3p Bmn-miRNA-399f Bmn-miRNA-444b.1 Bmn-miRNA-403e Bmn-miRNA-159 Bmn-miRNA-857	*Bacapa monnieri*	2019	Human genes	Bioinformatics analysis	Involvement in Nf-kB and MAPK pathway	[168]

### Plant miRNA transportation in mammals

**Fig. 3:**
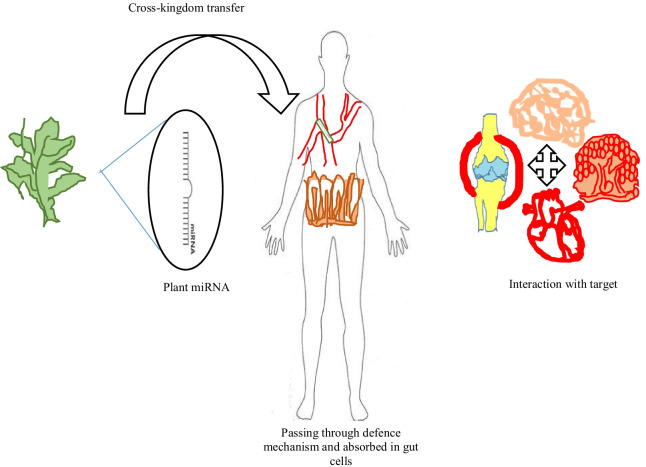
Plant miRNAs cross-defence mechanisms such as the blood–brain barrier. The RNA transporter protein aids in the absorption of miRNA through gut cells. SID1-2 promotes miRNA uptake and interacts with targets such as (cardiovascular disease, cancer cells, Alzheimer diseases, rheumatoid arthritis and so on)

Several issues remain unresolved regarding the transport of plant miRNA into an animal system [196]. In terms of plant miRNA stability, it may be jeopardised by a variety of factors such as enzyme degradation and the establishment of miRNAs in target cells [197]. Plant miRNAs are stabilised in mammalian cells by the 2′-O-me groups on the ribose of the last nucleotide [145]. As a result, the deterioration ratio of plant miRNAs is kept to a minimum. Several defence mechanisms, such as the blood–brain barrier (BBB) and other physiological barriers, influence plant miRNA absorption in mammals. As a result of plant miRNA absorption, the intestine's epithelial cells incorporate with miRNAs via various processes, plant miRNA reach gut cells, are delivered to specific physiological sub-compartments and different body systems, and modulate gene expression. The multispatial transmembrane proteins SID-1 and SID-2 promote siRNA uptake in *Caenorhabditis elegans*. A SID-1 could direct the passive diffusion of dsRNA via channel formation. SID-2 is required for environmental RNAi. Despite their extensive articulation, SID-1 and SID-2 are restricted to the apical membrane and expressed in the intestine, where they may play a role in the endocytosis of dsRNA from the lumen [198]. In mammals, an RNA transporter protein is present on the cell surface, which facilitates the transport of plant miRNA across the intestinal lumen (Fig. 3).

## In silico and in vivo approaches for plant miRNA absorption

Nutrichem2.0 is a plant-based food software that connects protein targets to FDA-approved drugs and small molecules found in plant-based nutrients. There are 428 drugs and 339 foods that have been shown to interact with protein targets. These in-silico approaches collect data on chemical bioactivity and allow for the comparison of activity concentrations between drugs and phytochemicals [199]. miRNA is still necessary to recognise in order to identify a potential transporter protein in mammalian cells for a plant. Synthesized plant miRNAs-34a, 143,145 act as tumour suppressor miRNAs in the in vivo model, reducing colon cancer by removing oncogenesis [200]. The turbulence surrounding the plant miRNA metabolism mechanism by mammalian liver cells revealed that miRNAs loaded with fluorescently labelled microvesicles were transported in target cells. miRNAs are protected from degradation by RNases after being packaged in microvesicles or exosomes. Phagocytosis and carrier-mediated regulation were used to transport these exosomes. Because milk is a bioactive food resource, a cow is the primary consumer, and bovine-milk-derived miRNA can travel the human intestinal tract via kinetic mechanism and reject surface exosome proteins. According to a recent pharmacokinetic study, cow milk acquired miRNA miRNA-29b and miRNA-200c to manage gene networking by targeting RUNX2 (runt-related transcription factor 2). Furthermore, these bovine-derived miRNAs aid gene modelling in human leukocytes, *in vitro* kidney cells, and mouse liver. However, the activation, stabilisation, and post-transcriptional mechanisms of miRNA remain a mystery [201].

**Fig. 4:**
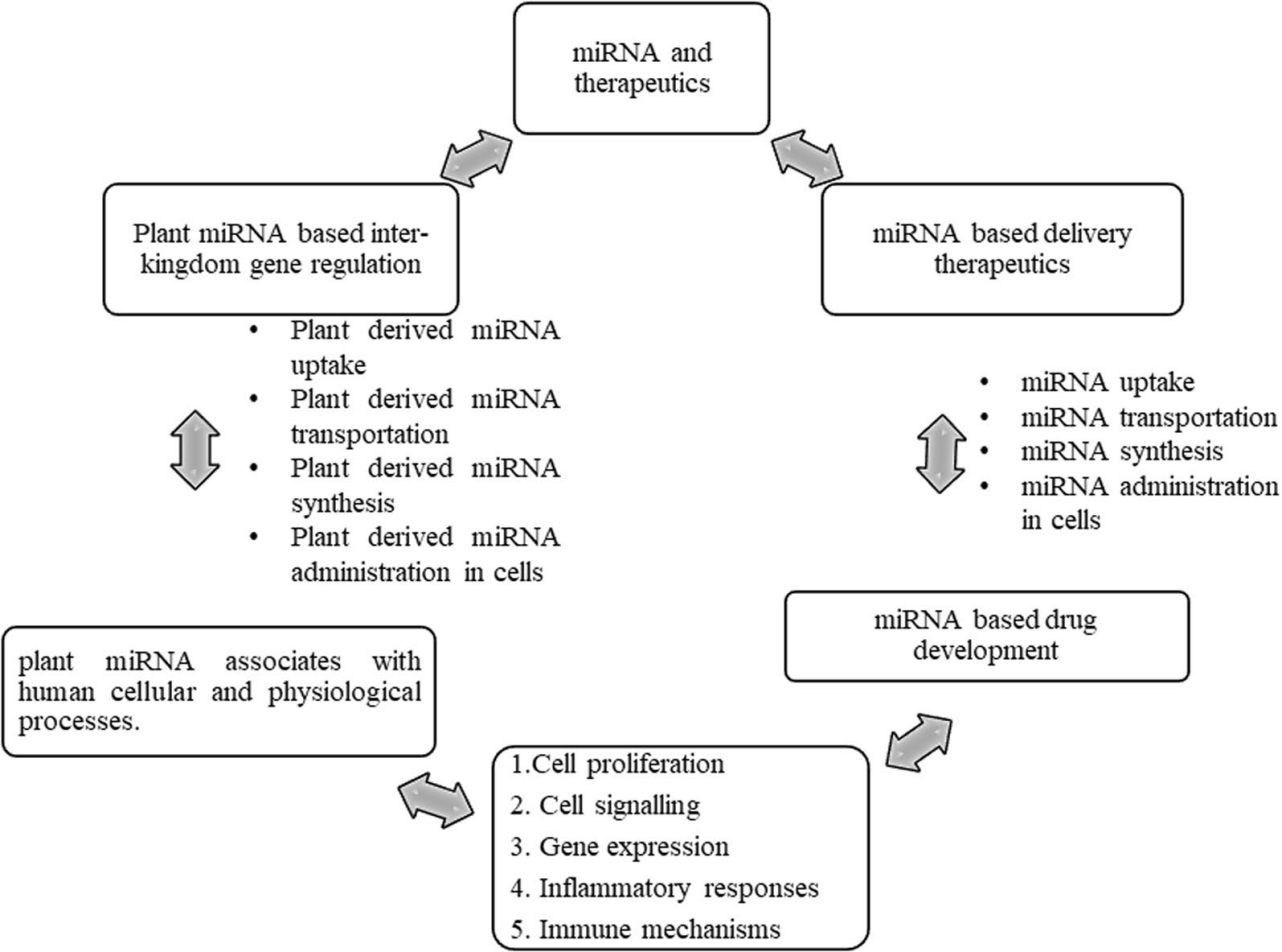
In miRNA-based delivery therapy, miRNA uptake, miRNA transportation, miRNA synthesis, and miRNA administration are all included. miRNA-based therapeutics are being developed to treat cell proliferation, cell singling, gene expression, inflammatory responses, and immunological processes. Furthermore, plant miRNA absorption, transportation, synthesis, and administration are all predicted for inter-kingdom investigations

Hypotheses are also generated regarding the significance and association of miRNA, as well as their potential maternal–fetal transfer for strong immunity and cell–cell communication. According to research, maternal breast milk contains a high concentration of miRNA. More than 65% of human breast miRNAs are involved in metabolic and immune processes. miRNA-181a and 155 coordinate B- and T-cell proliferation in order to improve adaptive immunity [202, 203]. As previously stated, miRNA-168a inhibits the low-density lipoprotein receptor adapter protein 1 (LDLRAP1), which inhibits cholesterol transport [190]. Furthermore, the author identifies the anti-viral property of honeysuckle miRNA-2911 as having an effect on influenza-affected mice models [204]. Many questions were raised as a result of the justification for plant miRNAs as nutritional mechanisms in mammals, such as post-transcriptional regulators (Fig. 4).

## Plant miRNA and SARS-CoV-2

COVID-19 has been confirmed in 281,808,270 cases in 223 countries, with 5,411,759 deaths, according to the global status as of December 2021. South-East Asia has 44,933,587 confirmed cases, making it the third most affected region after America and Europe [205]. SARS-CoV-2 is a coronavirus with a genome size of 26–32 kb that belongs to the β-subfamily identical to SARS-CoV and MERS-CoV. The SARS-CoV-2 virus was divided into four structural proteins: a spike, a membrane envelope, and a nucleocapsid [206]. 2019-nCoV's single-stranded RNA genome contains 29,891 nucleotides that encode 9889 amino acids [207]. miRNAs have the ability to inhibit messenger RNA translational activity and stability (mRNAs). These miRNAs play a role in a variety of cellular processes, including inflammation, cell cycle regulation, stress response, cell differentiation, migration, and apoptosis. The most effective interacting agents with coronavirus gRNA were identified as miRNA-4778-3p, miRNA-6864-5p, and miRNA-5197-3p. As a result, cellular miRNAs may be the best candidates for developing miRNA-based therapies for coronavirus diseases [208]. A cross-kingdom analysis reveals that plant miRNA may inhibit SARS-CoV-2 replication. miRNA-2911 from honeysuckle may be significantly absorbed in human serum, and in clinical studies, patients who received miRNA-2911 had a higher negative status than patients who did not receive miRNA-2911 treatment. This finding may shed light on the additional value of plant miRNA-based therapeutics, as well as their potential role in viral growth inhibition [209]. Despite several limitations, plant-derived miRNA therapeutics would establish a new promising area in plant-based therapeutics in the near future.

## Conclusions

Growing evidence suggests that miRNA have important biological functions in the cellular homeostasis. When miRNA regulation is disrupted, it can lead to the development of a variety of disease phenotypes. miRNAs have enormous clinical applications because they can be detected in blood, serum, tissues, and fine needle aspirate specimens (FNA). miRNA could be used as a non-invasive biomarker for a variety of diseases, and it can regulate cellular, metabolic, and physiological pathways in both normal and diseased conditions. The most appealing aspect of miRNA-based therapy is that a single miRNA can be used to target multiple genes that are dysregulated in diseases. Several plant miRNAs are also important in inter-kingdom gene regulation. As a result, the therapeutic value of plant-based miRNA is currently being researched for a variety of dysfunctions. If plant-based therapeutic miRNAs overcome checkpoints to become phyto-drugs, they will be less toxic, have precise target identification, and have a valid application to regulate various diseases. miRNA therapeutics and plant-based miRNA therapeutics are less explored in the current scenario, but in the near future, miRNA-based therapeutics will be a new hope for disease identification and regulation. In conclusion, miRNA have the potential to provide diagnostic, prognostic, and therapeutic targets. As the field develops, miRNA-based therapeutics may lead to the development of a new class of drugs for a variety of diseases.

## Data Availability

The references were used to gather all of the information in the manuscript.

## Abbreviations

miRNA: Micro RNA
ncRNAs: Non-coding RNAs
pri-miRNAs: Primary miRNAs
pre-miRNAs: Precursor miRNAs
mRNA: Messenger RNA
RBPs: RNA binding proteins
Ago: Argonaute family protein
EXP5/XPO5: Exportin 5
RISC: RNA-induced silencing complex
miRISC: MiRNA-induced silencing complex
CpG: Cytosine-phosphate-guanine
ADME: Absorption, distribution, metabolism and excretion
3′UTRs: 3′ Untranslational regions
CYP450: Cytochrome p450 family
DMEs: Drug metabolizing enzymes
DTs: Drug transporters
ABC: ATP binding cassette
SLC: Solute carrier
ABCB1/MDR1/P-gp: ATP binding cassette, subfamily B member 1
piRNAs: Piwi-interacting RNA
LNA: Locked nucleic acids
PMOs: Phosphorodiamidate morpholino oligonucleotides
PNA: Peptide nucleic acids
AMOs: Anti-miRNA oligonucleotides
PS: Phosphorothioate
2′-O-Me: 2′ Methyl
5′ NCR: 5′ Non coding region
HCV: Hepatitis C virus
CTCL: Cutaneous T-cell lymphoma
MF: Mycosis fungoides
DMD: Duchenne muscular dystrophy
CMV: Cytomegalovirus retinitis
cssDNA: Circular single-stranded DNA
EAE: Autoimmune encephalomyelitis
CRISPR: Clustered regularly interspaced short palindromic repeats
Cas9: CRISPR- associated protein 9
crRNA: CRISPR RNA
AAV: Adeno-associated viral
NPs: Nanoparticles
UMTD: Ultrasound targeted microbubble distraction
CSC: Cancer stem cells
DLC1: DICER-LIKE 1
HEN1: HUA enhancer
UPE: Unpaired energy
HEPG2: Human hepatoma cell line
ESTs: Express sequence tags
LDLRAP1: Low density lipoprotein receptor adaptor protein
LPS: Lipopolysaccharide
Poly I:C: Polyinosinic: polycytidylic
BBB: Blood–brain barrier
SID-1: Systemic RNA interference deficiency 1
RUNX2: Runt-related transcription factor 2
